# Contextually conspicuous species are more likely to be venomous: Habitat‐dependent aposematic colouration found throughout snakes

**DOI:** 10.1111/1365-2656.70351

**Published:** 2026-10-04

**Authors:** Amy Duclaux, Michel Dugon, Kevin Healy

**Affiliations:** ^1^ Zoology Department, School of Natural Sciences University of Galway Galway Ireland

**Keywords:** Aposematism, Colour, Macroecology, Serpentes, Venom

## Abstract

1. Many animals use conspicuous colours to advertise their unprofitability to potential predators, a phenomenon known as aposematism. For example, conspicuous banded patterns in snakes are often highlighted as aposematic signalling of their venomous nature. However, aposematic colouration needs to be analysed while considering a species' environmental context, seen as species which seem conspicuous in isolation may be well camouflaged within their natural environment.
2. Here, we collate images for 458 snake species from iNaturalist and use a novel metric quantifying the degree of conspicuousness of each species compared with its natural context to test the relationship between conspicuousness and the presence, potency and defensive properties of venom.
3. We find that venomous species are significantly more contextually conspicuous compared with non‐venomous species. However, while these results provide support for the link between the evolution of aposematism in relation to venom, we do not find an association with the potency or the pain‐inducing capacity of venoms with the data used in this study, indicating that these traits may not be important drivers of the evolution of aposematism in snakes.
4. Our novel approach demonstrates how incorporating the environmental context of a species can shed light on our understanding of the evolution of conspicuous colouration.

## Graphical abstract

Conspicuous colouration signals venomous nature throughout snakes.

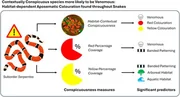

## 1 INTRODUCTION

Aposematism, the use of conspicuous signals which evolved under natural selection pressures to warn potential predators of a prey's unprofitability (Caro & Ruxton, 2019; Poulton, 1890; Wallace, 2016), is widespread across the animal kingdom. These signals are typically associated with traits which aid in defence such as venoms (Eisner et al., 2005), spines (Speed & Ruxton, 2005) and alkaloid toxins (Santos et al., 2003), and are notable by how the signal often dramatically increases conspicuousness. In direct contrast to avoiding a predator's attention through camouflage, aposematism requires the clear signalling of visual information towards potential predators, which in turn can lead to predators learning specific warning cues and the evolution of innate avoidance behaviour (Kang et al., 2016; Mappes et al., 2005). However, while strong patterns and coloration may seem to indicate aposematism, such potential signals are not always conspicuous within the context of a species' natural habitat.

Conspicuous colouration is habitat‐dependent. For example, while the monarch butterfly (*Danaus plexippus*) is thought to use its vivid orange and black colouring to signal its toxicity to avian predators, these same hues also allow it to blend in with the autumnal foliage (Brower, 1988). However, while the conspicuousness of some colour patterns may be context‐dependent, others such as red and yellow are likely to be aposematic in most natural habitat types (Emberts & Wiens, 2022), such as seems to be the case with the bright red seen on many ladybug species (*Coccinellidae*; Holloway et al., 1991) and vivid yellow spots sported by fire salamanders (*Salamandra salamandra*; Brandon & Huheey, 1975; Caspers et al., 2020). While several studies have tested aposematic signalling in relation to its environmental context using various methodologies, including using plastic models in the field (Preißler & Pröhl, 2017; Valkonen et al., 2011; Wüster et al., 2004), or human observer scores (Arbuckle & Speed, 2015; Goldenberg et al., 2025), large‐scale analysis which directly compare species coloration to their habitat is still lacking, likely due to the difficulty of compiling large‐scale data of species within their natural context.

Citizen science initiatives such as iNaturalist (iNaturalist, 2025) allow for large‐scale image data availability of accurately identified species in their natural habitat, with citizen science photographs found to be reliable for large‐scale comparative analysis of various traits, including foraging ecology (Silva et al., 2024), habitat type (Fu et al., 2024) and colour (Laitly et al., 2021). With the rise of the quality and quantity of images being uploaded, they offer a valuable resource for extracting data such as species colouration (Mason et al., 2025). By focusing on taxonomic groups with wide coverage in iNaturalist, such data have been used to test aspects of colour variation across species including polymorphism in lace monitors (*Varanus varius*; Farquhar et al., 2023) and European adders (*Vipera berus*; Jansen et al., 2025), thermoregulation in green anoles (*Anolis carolinensis*; Price et al., 2025) and sexual dichromatism throughout anole lizards (*Anolis*; Westeen et al., 2025). One large charismatic group of animals which makes an ideal model system for testing conspicuous aposematic colouration and are also very well‐documented on citizen science applications are snakes.

Snakes (suborder *Serpentes*) display a wide variation in their coloration, the environments they inhabit and the degree to which they can use venom as a defensive mechanism. They employ a range of colours, some of which are assumed to be aposematic, such as the many banded species of conspicuous coral snakes (*Micrurus*; Mouy, 2024; Smith, 1975, 1977) and their harmless mimics (Brodie III & Janzen, 1995), while others, such as Lotiev's viper (*Vipera lotievi*), clearly display colours adapted for camouflage (Figure 2). Among snakes, venom potency is also highly variable, with some snake venoms also found to have defensive toxins that specifically cause pain (Bohlen et al., 2011; Fernández et al., 2015; Kazandjian et al., 2021; Zhang et al., 2017). This variation in the capacity to use venom in defence, along with the range of coloration they display, makes snakes an ideal group to test the link between venomous traits and aposematic signalling. Furthermore, while there has been mixed evidence supporting the relationship between the degree of aposematic signalling and potency, with support for a negative association in *Epipedobates* poison frogs (Darst et al., 2006) and a positive association in dendrobatid frogs (Summers et al., 2003), there is no association found in snakes (Allen et al., 2013), which may be due to the missing component of environmental context.

While we expect the presence of defensive traits to be the primary driver of aposematic conspicuousness, several aspects associated with specific ecologies are also likely to be important drivers. For example, daily temporal activity is strongly linked to the evolution of conspicuous colouring, with diurnal species such as lizards and birds evolving conspicuous colours for conspecific sexual signalling (sexual dichromatism) while the evolution of aposematic colours is associated with animal groups which were ancestrally nocturnal, such as amphibians and snakes, which instead of evolving bright colours to attract a mate may have done so to deter diurnal predators (Emberts & Wiens, 2022). The physical nature of a habitat is also potentially important in influencing warning signals as predation risk is expected to differ between terrestrial, arboreal and aquatic habitats due to the visual orientation of potential predators (Senter, 1999). For example, compared with the two‐dimensional nature of terrestrial habitats, arboreal and aquatic species are expected to have higher predation risks from both above and below (Martins et al., 2008; Pawar et al., 2012; Senter, 1999). Finally, although snakes may use aposematic colouration to deter predators, they themselves are predators to a wide range of prey and may also be under selection to remain concealed to those species. Snakes feed on a variety of prey items (O'Shea, 2018) all of which have different visual systems, ranging from achromatic visual systems in soft‐bodied invertebrates (Chernorizov & Sokolov, 2010), to dichromatic visual systems in rodents (Bowmaker, 2008; Jacobs et al., 2001) and complex tetrachromatic visual systems in birds (Bowmaker, 2008; Hart & Hunt, 2007). Hence, depending on the visual system of their prey, some snake species would be expected to be less constrained on their aposematic colouration.

While the presence of aposematic signalling has been demonstrated in certain snake groups such as coral snakes (Mouy, 2024; Smith, 1975, 1977) and European vipers (Valkonen et al., 2011; Wüster et al., 2004), there has not been a group‐wide study which has established a relationship between conspicuous warning signals and venomousness in snakes (Allen et al., 2013). Here, we test the link between aposematic warning colouration and properties of a snake species' venom, including its presence, potency and ability to induce pain. We also test if specific patterning, such as banded patterns, and behavioural and ecological factors, such as daily activity, habitat type and prey visual system, influence conspicuous colouration in snakes. We predict that more habitat‐dependent conspicuously coloured snakes are more likely to be venomous, have comparatively less potent venom due to learnt avoidance behaviour from predators and possess defensive, pain‐inducing venom.

## 2 METHODS

### 2.1 Data collection

To test our hypothesis, we collated available data for conspicuous colouration, venomous traits, daily activity, habitat type and prey visual system for 458 different Colubroidae snake species, with representatives from the *Colubridae* (112 species), *Cylindrophiidae* (1 species), *Dipsadidae* (95 species), *Elapidae* (99 species), *Lamprophiidae* (39 species), *Natricidae* (23 species), *Sibynophiidae* (3 species) and *Viperidae* (117 species) families. Species selection was based on curating the largest possible database of taxa for which venom (presence, potency and defensive properties), image and ecological (daily activity, habitat type and diet) data were available.

We collected data relating to whether a snake species had venomous toxins present or not by first collating venomous/non‐venomous categorical data from the ReptTraits database (Oskyrko et al., 2024). As this categorisation is based on medical significance and not the presence of orally delivered toxic secretions *stricto* sensu, we further screened the data and added snake species to the ‘venomous’ category if the literature supported that either (1) they possessed Duvernoy's glands (Taub, 1967), or (2) were found to produce toxins linked with predation, such as three‐finger toxins (Dashevsky et al., 2018), or (3) otherwise showed evidence of being able to incapacitate prey via the oral delivery of toxins. In this study, we categorised 353 snake species as ‘venomous’ and 104 species as ‘non‐venomous’. Potency measures for venom were collated from Healy et al. 2019 (Healy et al., 2019) as intravenous LD_50_ (Lethal Dose 50%) values. While historical LD_50_ measures are typically measured from mammalian lab models such as mice (*Mus musculus*), where available, we also used measurements taken from other animal models which more closely reflect specific snake species diet, such as fish models for the aquatic olive sea snake (*Aipysurus laevis*) or bird models for the arboreal brown tree snake (*Boiga irregularis*). Measures relating to the pain‐inducing properties of a species' venom were collated from Ward‐Smith et al. (2020) which was based on ratings of rapid pain onset from human snake bite victims. Although this measure is biased towards the experience of human pain, it is likely to capture general mammalian predator pain experience as pain‐inducing toxins, such as phospholipase A_2_ (PLA_2_), are known to activate mammalian sensory neurons in general and cause enhanced pain (Kazandjian et al., 2021).

Image data used to quantify conspicuousness and colour (red and yellow percentage coverage) data were collected from iNaturalist (iNaturalist, 2025). For each species of Colubroidae present within the iNaturalist database, we selected those with CC‐BY‐NC, CC‐BY or CC0 copyright licencing and excluded any images where only small segments of the snake was visible (for example, images of just the head), where the individual was clearly not alive (such as roadkill) and images where the snake was clearly not in its typical habitat (for example, individuals on tarmacadam roads). From these images, we selected five at random for each species. Species with less than five images available were not included. For each image which met our criteria, the snake and background were separated by clipping, using the Clip Studio Paint software (Clip Studio Paint, 2025), and saved as separated PNG files. Separating a single image into the snake and its environment allows us to quantify the disparity in colouration between the specimen and its habitat using the R package ‘colordistance’ (Weller & Westneat, 2019) in R 4.5.1 (R Core Team, 2012), which generates a single colour distance matrix (or CDM) value which quantifies the difference occupied in the three‐dimensional red green blue (or RGB) colour space between two images (Figure 1). Larger CDM values represent greater colour disparity between the colour spaces present in the snake and those present in its environment. We also used the ‘countcolors’ package (Weller, 2019) in R 4.5.1 (R Core Team, 2012), which allowed us to quantify the percentage coverage of specific colours; in our case, the percentage coverage of red and yellow in snake species. We followed a similar approach to Delhey et al. (2023) and defined the ranges of our colours of interest in the RGB space. We defined red at an RGB centre of c(1, 0, 0) with a range of 0.3 and our centre for yellow was set at c(1, 0.8, 0.2) with a range of 0.15. Based on the image data collected, species were either categorised as having banded patterning or not, with banded patterning being strictly allocated to species which have very distinct and highly contrasting banding, such as is seen in many *Micrurus* species.

**FIGURE 1:**
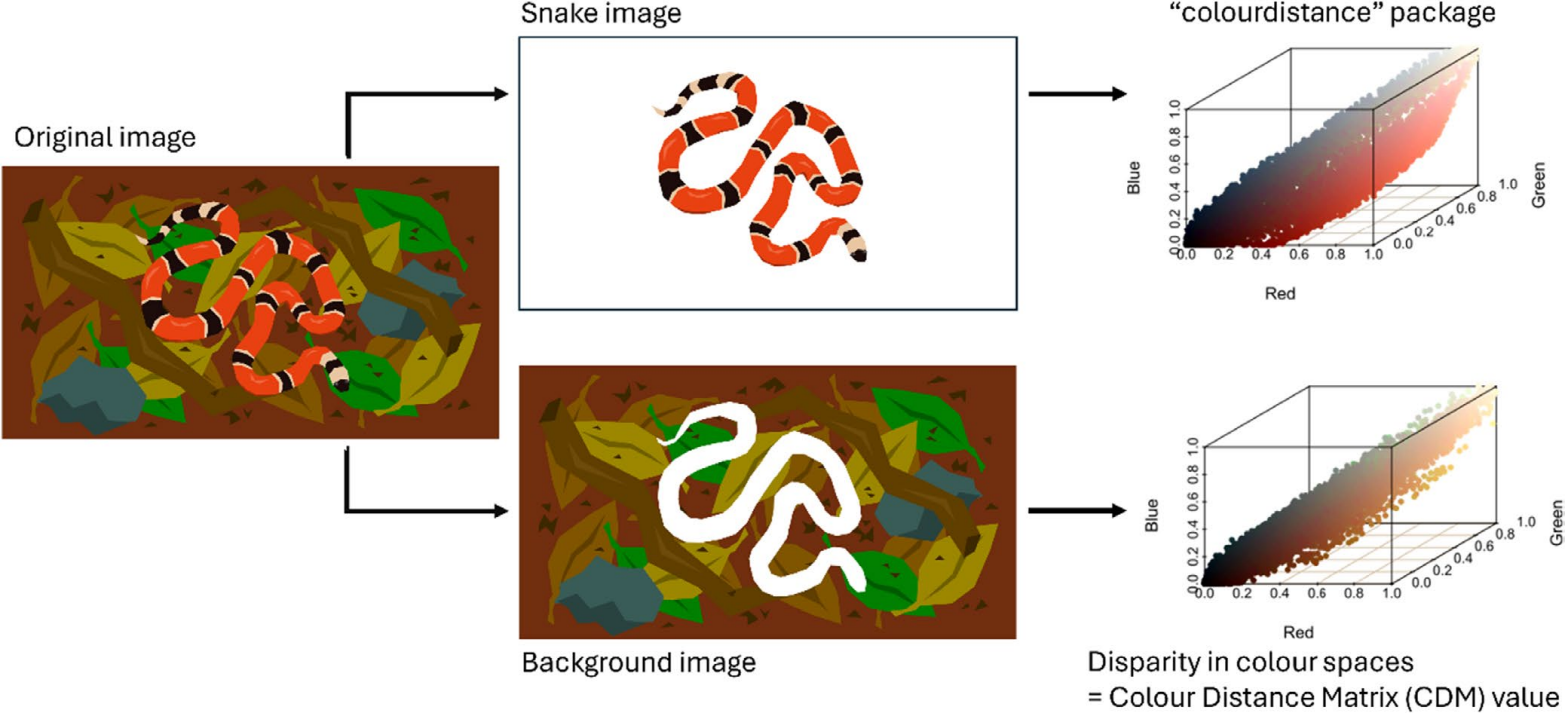
Image editing process for quantifying relative conspicuousness of snake species relative to its environment. The original image is split into two separate images: An image with only the snake and an image with only the image background (the snake's environment). The ‘colourdistance’ package in R obtains the RGB colour space of each image and then calculates the disparity in colour spaces occupied by both images; the more conspicuous the snake is compared with its environment, the greater the colour distance matrix (or CDM) value. Illustrations by Amy Duclaux.

We collated daily activity and habitat type from the IUCN Red List (IUCN, 2025) species description, where daily activity was categorised as either diurnal, nocturnal or both (cathemeral) and habitat type was categorised as terrestrial, arboreal or aquatic. Due to smaller sample sizes for semi‐arboreal (*n* = 47 species) and semi‐aquatic (*n* = 37 species) habitat type, we categorised semi‐arboreal species as arboreal and semi‐aquatic species as aquatic. We also collected diet data from the IUCN Red List (IUCN, 2025). Based on the visual system of the prey item a species fed on, the prey visual system was either categorised as simple or complex; diets which contained birds, lizards, amphibians (including tadpoles) or fish were considered to have prey with complex visual systems due to the trichromatic and tetrachromatic visual systems of these groups (Bowmaker, 2008), with species feeding on soft‐bodied invertebrates (such as snails, slugs and earthworms), amphisbaenians, other snakes, mammals and rodents were considered to have prey with simple visual systems due to their achromatic and dichromatic visual perceptions (Bowmaker, 2008; Chernorizov & Sokolov, 2010; Jacobs et al., 2001). Three species, the Egg‐eating sea snake (*Emydocephalus annulatus*), the Colombian longtail snake (*Enuliophis sclateri*) and the Ceylon wolf snake (*Lycodon carinatus*), which possess specialised egg diets, were removed from the study. When data relating to daily activity, habitat type or diet was missing from the IUCN Red List species description, we used the Reptile Database (The Reptile Database, 2025), the Book of Snakes by O'Shea (2018) and the WCH Clinical Toxinology Resources database (WCH Clinical Toxinology Resources, 2025) as additional sources in order to maximise our species sample size. Due to the nature of this study, we did not require ethical approval.

### 2.2 Data analysis

In order to test our hypothesis, we fit the conspicuousness measures of CDM, red percentage coverage and yellow percentage coverage as the response variables in separate models; these three conspicuousness proxies capture the intensity of natural selection pressure on aposematic signalling. In these models, we included the venom function measure as an explanatory factor with models including either the measure of the presence of venomous toxins (present, absent), LD_50_ (mg) or pain‐inducing effects (present, absent). Due to different samples sizes for these venom function measures, we included them in separate models, resulting in nine models, three measures of venom function for each of the three response variables measuring conspicuousness levels. We ran an extra model to test whether species with higher degrees of red or yellow percentage coverage were more likely to be contextually conspicuous, with CDM value as the response variable. To account for Batesian mimicry, which is when a harmless species mimics the colouration and patterning of a harmful one (Bates, 1862), we reran an extra three presence of venomous toxins (present, absent) models and removed 15 snake species which mimic the banded appearance of highly venomous species such as coral snake (*Micrurus*) mimics (*Atractus latifrons*, *Oxyrhopus guibei*, *O. trigeminus* and *Simophis rhinostoma*; Davis Rabosky et al., 2016; Fogden & Fogden, 1974), Asian coral snake (*Calliophis*) mimics (*Cylindrophis ruffus*; Fogden & Fogden, 1974) and krait (*Bungarus*) mimics (*Lycodon aulicus, L. flavozonatus, L. futsingensis, L. rosozonatus, L. rufozonatus, L. striatus, L. travancoricus, L. zawi* and *Oligodon arnensis*; Whitaker et al., 2004); we also removed *Coronella austriaca* which mimics the zig‐zag patterning of European viper species (*Vipera*; Valkonen et al., 2018). In all models, we included the explanatory variables of banded patterning (present, absent), daily activity (diurnal, nocturnal, cathemeral), habitat type (terrestrial, arboreal, aquatic) and prey visual system (simple, complex). The structure of the models is as follows:

- Conspicuousness (CDM value) ~ Venom function measure + Banded patterning + Daily activity + Habitat type + Prey visual system
- Red percentage cover ~ Venom function measure + Banded patterning + Daily activity + Habitat type + Prey visual system
- Yellow percentage cover ~ Venom function measure + Banded patterning + Daily activity + Habitat type + Prey visual system
- Conspicuousness (CDM value) ~ Red percentage cover + Yellow percentage cover

We fit all models using Bayesian phylogenetic mixed models using the MCMCglmm package (Hadfield, 2010) and used the animal model which includes a term that accounts for non‐independence due to common descent and calculated the *h* ^2^ value which is analogous to Pagel's lambda, with higher values indicating higher phylogenetic signals (Pagel, 1999). To include uncertainty related to the phylogenetic tree, we used the mulTree package (Guillerme & Healy, 2014) to run each of the above models across 100 random phylogenies from the distribution of phylogenies from Tonini et al. (2016). We also included a random term at the species level to account for variation within species. We used expanded uninformed priors and chain parameter settings that resulted in all estimates having effective sample sizes above 1000. We ran three chains for each model and checked for convergence using the Gelman–Rubin statistic (Andrew & Donald, 1992). Model estimates were considered significant when the 95% credible intervals (CI) did not cross zero. All analyses were carried out in R 4.5.1 (R Core Team, 2012).

## 3 RESULTS

Our analysis included 458 Colubroidae snake species with conspicuousness ranging from CDM values of 0.03–0.81 (range of 0.78); with the well camouflaged nose‐horned viper (*Vipera ammodytes*) having a CDM value of 0.03 to the much more contextually conspicuous banded ornate sea snake (*Hydrophis ornatus*) having a CDM value of 0.81. Red and yellow percentage coverage also varied greatly, with red percentage cover reaching as high as 69.5% in Reinhardt's burrowing snake (*Apostolepis assimilis*) and yellow percentage cover reaching 46.6% in the yellow‐bellied sea snake (*Hydrophis platurus*; Figure 2).

**FIGURE 2:**
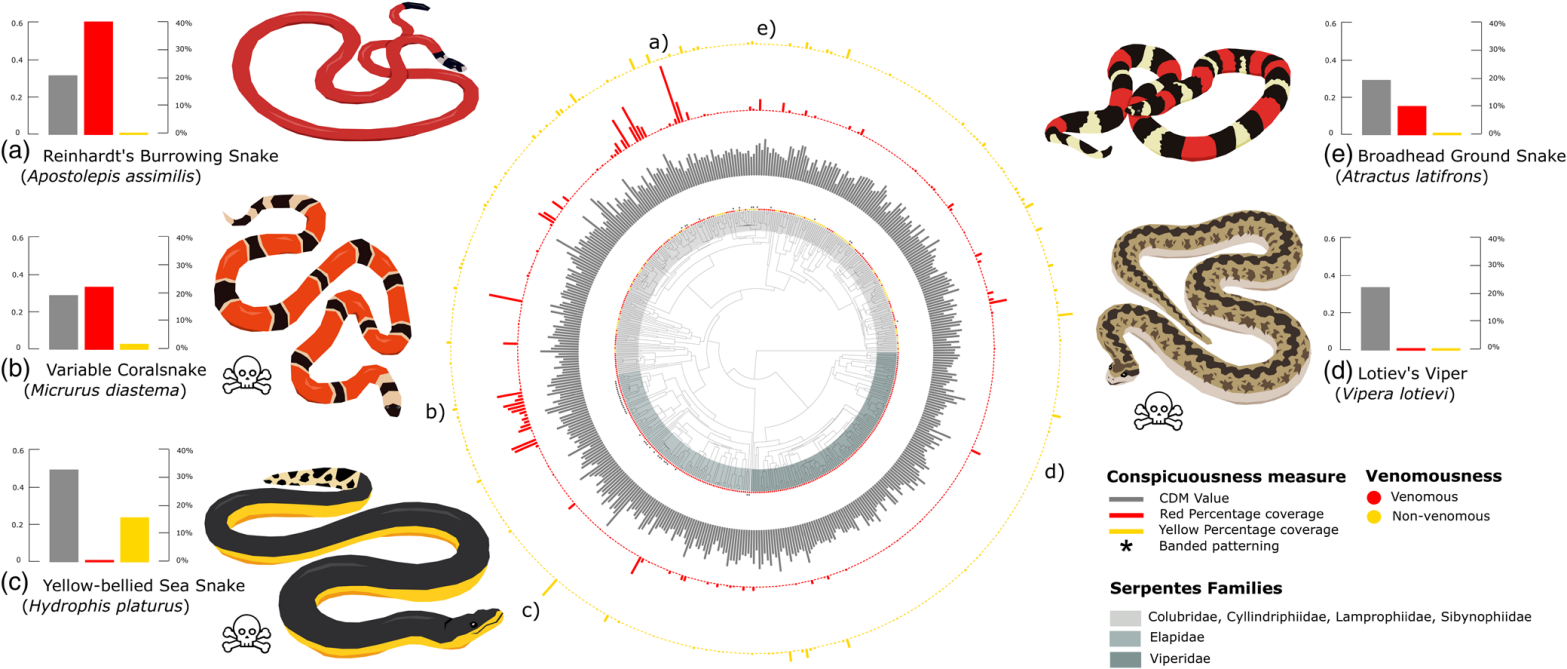
Phylogeny representing the diversity of conspicuousness (measured with CDM values, red percentage coverage and yellow percentage coverage; average values presented on phylogeny) displayed in the 458 snake species included in our study spanning major Colubroidae families (Colubridae, Cylindriphiidae, Lamprophiidae, Sibynophiidae, Elapidae, Viperidae). Species highlighted to illustrate the diversity of conspicuousness found in our study are (a) Reinhardt's burrowing snake (*Apostolepis assimilis*), (b) variable coral snake (*Micrurus diastema*), (c) yellow‐bellied sea snake (*Hydrophis platurus*), (d) Lotiev's viper (*Vipera lotievi*) and (e) broadhead ground snake (*Atractus latifrons*). Illustrations by Amy Duclaux.

Overall, we find that snake species which possess venom are associated with significantly higher levels of conspicuousness compared with non‐venomous species, based on whether the posterior estimate credible intervals contained zero, in both our analysis including mimic species (Table 1) and in the analysis excluding mimic species (Table S9). We also find that snake species displaying greater degrees of red and yellow are significantly more contextually conspicuous in their natural habitat based on CDM values (Table 2, Figure 3) and that species which possess venom are associated with a higher percentage of red colouring compared with species which do not (Table S10). However, while we find that the presence of venom predicts an increase in conspicuousness, we do not find any support for an association between the potency or pain‐inducing properties of venom and levels of conspicuousness in snakes (Tables S4–S9).

**TABLE 1:** Mode of Posterior Estimates and 95% credible intervals (CI) for the posterior distributions for the conspicuousness model which tests the relationship between conspicuousness (measured with CDM values) and the presence of venomous traits (present, absent), along with banded patterning, daily activity, habitat type and prey visual system.

	Mode	Lower 95% CI	Upper 95% CI
Fixed terms			
Intercept	0.223	0.157	0.287
Venomous (Present)	**0.023**	**0.005**	**0.040**
Banded patterning (Present)	0.016	−0.006	0.038
Daily activity (Cathemeral)	−0.011	−0.030	0.007
Daily activity (Nocturnal)	−0.013	−0.028	0.003
Habitat type (Arboreal)	−0.001	−0.018	0.016
Habitat type (Aquatic)	0.008	−0.017	0.033
Prey visual system (Complex)	0.014	−0.003	0.032
Random terms			
Residual variance	0.015	0.014	0.016
Phylogenetic variance	0.003	0.002	0.005
*h* ^2^	0.165		

*Note*: The explanatory variables include the presence of venomous traits; banded patterning; daily activity; habitat type and prey visual system. The random terms associated with phylogenetic relatedness (Phylogeny (*h* ^2^)), and residual variation (Residual) are also presented. Significant values are deemed to be those with 95% of the posterior estimate above or below zero. Overall, there are 458 species in this model (including mimicking species) run across 100 randomly selected phylogenies. Model estimates were considered significant when the 95% credible intervals (CI) did not cross zero.

**TABLE 2:** Mode of posterior estimates and 95% credible intervals (CI) for the posterior distributions for the conspicuousness model which tests the relationship conspicuousness (measured with CDM values), red percentage coverage and yellow percentage coverage.

	Mode	Lower 95% CI	Upper 95% CI
Fixed terms			
Intercept	0.240	0.180	0.299
Red percentage coverage	**0.001**	**0.0001**	**0.002**
Yellow percentage coverage	**0.006**	**0.004**	**0.008**
Random terms			
Residual variance	0.015	0.001	0.005
Phylogenetic variance	0.003	0.001	0.005
*h* ^2^	0.169		

*Note*: The explanatory variables include red and yellow percentage coverage. The random terms associated with phylogenetic relatedness (Phylogeny (*h* ^2^)), and residual variation (Residual) are also presented. Significant values are deemed to be those with 95% of the posterior estimate above or below zero. Overall there are 458 species in this model run across 100 randomly selected phylogenies. Model estimates were considered significant when the 95% credible intervals (CI) did not cross zero.

**FIGURE 3:**
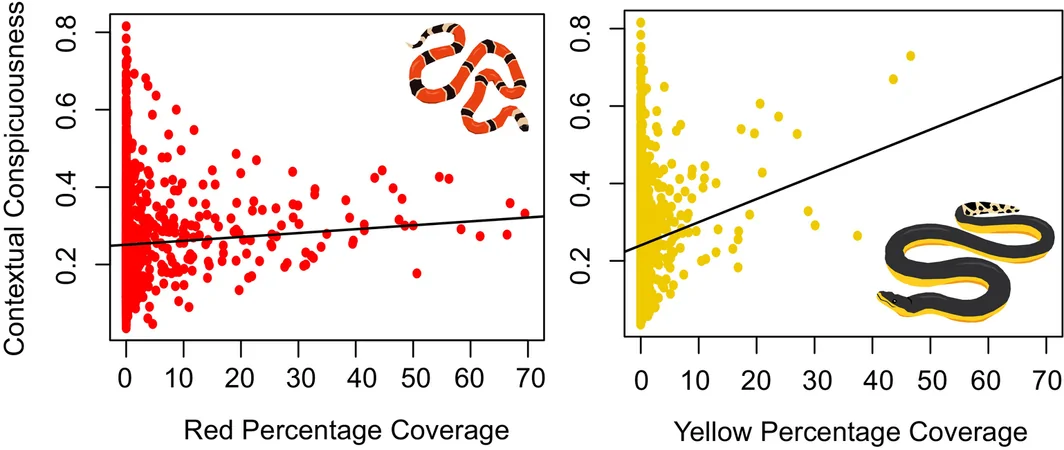
Plot representing the relationship between contextual conspicuousness, red and yellow percentage coverage. We find that contextual conspicuousness is related to increased degrees of red and yellow colouration in snakes, with snake species displaying higher levels of red and yellow in their colouration being significantly more contextually conspicuous in relation to their natural habitat. Illustrated are the variable coral snake (*Micrurus diastema*) and the yellow‐bellied sea snake (*Hydrophis platurus*), respectfully. Illustrations by Amy Duclaux.

As expected, we find that snake species with banded patterning have significantly higher percentage coverage of both red (Tables S1, S7 and S10) and yellow (Tables S5 and S8) but not with overall conspicuousness as measured by CDM values (Table 2; Table S9). Cathemeral daily activity was also associated with higher yellow percentage coverage (Tables S2 and S11) in addition to arboreal habitat type (Tables S2, S8 and S11) and aquatic habitat type (Table S2). We found no association between the visual system of a snake's prey and overall conspicuousness or percentage coverage of red or yellow.

Finally, we found *h* ^2^ values of 0.086, 0.065, 0.077 and 0.065 indicating a weak phylogenetic signal for conspicuousness (Table 1, Tables S3, S6 and S9), *h* ^2^ values of 0.508, 0.001, 0.0002 and 0.503 indicating a more variable/very weak phylogenetic signal for red percentage coverage (Table S1, Tables S4, S7 and S10) and *h* ^2^ values of 0.0002, 0.003, 0.0006 and 0.0002 indicating a very weak phylogenetic signal for yellow percentage coverage (Tables S2, S5, S8 and S11).

## 4 DISCUSSION

Here, we show support that snake species which possess venomous traits are more conspicuous in their natural setting when compared with those which do not. These findings indicate that possessing venom is significantly associated with displaying contextually conspicuous aposematic warning signalling; however, our findings show little support that such displays are linked with venom potency or venom possessing defensive pain‐inducing toxins, suggesting that while the presence of venom may select for aposematic signalling, these specific venomous traits are not strong selectors for such signalling in snakes.

While previous analyses have highlighted the potential link between venomous traits and conspicuous aposematic coloration, significant support for this relationship has not previously been demonstrated (Allen et al., 2013). By incorporating the context of snake coloration, our analysis offers such support, highlighting that traits that can be used in defence, such as venom, are linked with the evolution of increased conspicuousness. Our results point to the potential importance of incorporating environmental context in studies of the evolution of colour patterns at large, macroecological scales. For example, studies looking at sexual signalling have focused primarily on images of museum specimens (Cooney et al., 2022) and scientific illustrations (Dale et al., 2015) but do not typically account for the environmental context of such coloration, potentially masking evolutionary patterns that we demonstrate here with the link between venom and aposematism.

We find that red, a colour often associated with aposematic warnings, was also significantly associated with the presence of venom. While a previous study by Allen et al. (2013) failed to establish the expected link between specific conspicuous colours and venom in snakes (Allen et al., 2013), this may have been partially due to only North American and Australian species being included in the study. Here, we find the anticipated association between higher proportions of conspicuous red colouring on the body and the presence of venom in snakes. Red is traditionally associated with warning signals in many terrestrial animal species and has been linked to aposematic signalling in various poisonous and venomous groups of animals such as dart frogs (Lorioux‐Chevalier et al., 2023; Maan & Cummings, 2012; Roberts et al., 2022), ladybird beetles (Holloway et al., 1991) and certain groups of snakes such as coral snakes (Mouy, 2024; Smith, 1975, 1977). Here, we find that red is likely to be a more general aposematic signal in snakes, with increased red colouring also shown to be associated with more contextually conspicuous species as measured using CDM values in our analysis. As expected, we also find that banded patterns were also associated with higher degrees of red. While previous studies have shown the association between conspicuous red colouration and banded patterning in snakes, such as in coral snakes *Micrurus* and *Micruroides* (Brodie III & Janzen, 1995; Davis Rabosky et al., 2016; Moore et al., 2020; Mouy, 2023; Savage & Slowinski, 1992), we show that this relationship between conspicuous red colouration and banded patterning is a general association throughout *Colubroidae* snakes.

Although yellow colouration is often associated with aposematic signals in many animal groups with defensive traits such as venomous wasps (Kauppinen & Mappes, 2003), poisonous salamanders (Brandon & Huheey, 1975; Caspers et al., 2020) and spiny tenrecs (specifically the lowland streaked tenrec, *Hemicentetes semispin*; Stankowich & Campbell, 2016), we did not find it to be associated with venomous traits in snakes. However, we did find that higher coverage of yellow is influenced by other ecological traits in snakes. We found that arboreal snake species were more likely to have higher percentage coverage of yellow, which is likely due to the common use of countershading camouflage (Ruxton et al., 2004), where darker green dorsal colours are often associated with a paler off‐white underbelly, with transitional yellows between the dorsal and ventral sides, such as in the bamboo viper (*Trimeresurus albolabris*; O'Shea, 2018). On the other hand, we also found some evidence that aquatic species were also more likely to sport higher yellow percentage cover, which could potentially be for aposematic purposes. Yellow may be a particularly effective colour for communication in aquatic settings, as shorter yellow colour wavelengths can penetrate deeper into the water column compared with longer red ones, making red a more inconspicuous colour in aquatic settings compared with yellow (Johnsen & Sosik, 2004).

While we found an overall relationship between conspicuousness and the possession of venom, we did not find support for the relationship with specific venom traits such as venom potency or its ability to induce pain. While it might be expected that species with milder venoms may be more likely to demonstrate aposematic coloration due to the increased chances of predators learning to avoid warning signals through experience (Kang et al., 2016; Mappes et al., 2005), we do not find evidence for this link. We also found no clear support between conspicuousness and defensive venomous properties, such as a venom's ability to induce rapid pain (Ward‐Smith et al., 2020). Our results are similar to previous analysis of warning signals in snakes (Allen et al., 2013); however, this study relies on human subjective measures of pain which, while they are likely to capture a venom's ability to inflict pain on mammalian predators, may overlook the capacities of venom to impart defensive outcomes on non‐mammal predators, such as birds and fish. Future approaches focusing on the presence of known pain‐inducing toxins specific to potential predator targets, such as the MitTx toxin, BomoTx myotoxin and phospholipase A_2_ toxins (Bohlen et al., 2011; Fernández et al., 2015; Kazandjian et al., 2021; Zhang et al., 2017), may offer clearer associations between defensive properties and aposematism.

While we found a link between yellow colouration, aquatic and arboreal habitat types, we did not find any ecological factors that influenced conspicuousness as measured using CDM values. While we might have expected higher levels of conspicuousness in nocturnal species, which may rely more on conspicuous warning colours in order to deter predators while resting during daylight hours (Emberts & Wiens, 2022), or diurnal species, which are potentially exposed to visually oriented predators (Brandon & Huheey, 1975), we do not find support for an association between a snake species' activity patterns and its level of conspicuousness or red and yellow coverage. It is also noteworthy that we did not find any relationship between conspicuous colouration and the prey visual system a snake species feeds on. Snakes which feed on prey with achromatic or dichromatic visual systems might have been expected to be more conspicuously coloured since they may not have had to rely on camouflage as much as those preying on species with more complex trichromatic or tetrachromatic visual perception (Bowmaker, 2008). While not tested here, it could be the case that predator and not prey visual systems are more important in driving conspicuousness in snakes; for example, a recent study showed that avian predation heavily impacted UV colouration in snakes (Crowell et al., 2024). As highlighted using moth models, aposematic colouration is more strongly driven by predation intensity compared with other factors such as illumination (Medina et al., 2025), indicating that quantitative measures of predation risk would particularly aid in testing the drivers of warning signals in snakes.

Overall, our large‐scale study spanning 458 globally distributed *Colubroidae* snake species shows that conspicuous aposematic colouration is linked to the presence of venom but not to other functional traits of venom, and that red and yellow colouration are considered contextually conspicuous in snakes. The use of large citizen driven databases, such as iNaturalist, can offer the scale to test general drivers of colour evolution; however, studies focusing on more specific aspects, such as behaviour associated with warning signals, may offer further insights into potential links with defensive traits of venom and aposematic signalling. Our study also highlights the importance of incorporating the context of a species' natural environment when measuring levels of conspicuousness, with such an approach likely to offer further insights into other aspects of the evolution of conspicuous signalling, such as sexual dichromatism (Emberts & Wiens, 2022).

## AUTHOR CONTRIBUTIONS

Amy Duclaux and Kevin Healy conceived the ideas and designed methodology; Amy Duclaux collected the data; Amy Duclaux, Kevin Healy and Michel Dugon analysed the data; Amy Duclaux and Kevin Healy led the writing of the manuscript. Amy Duclaux produced the figures. All authors contributed critically to the drafts and gave final approval for publication.

## FUNDING INFORMATION

This study was funded by Research Ireland (Taighde Éireann) to Amy Duclaux.

## CONFLICT OF INTEREST STATEMENT

The authors declare no conflicts of interest.

## Supporting information


**Figure S1.** Phylogeny representing the diversity of conspicuousness (measured with CDM values, red percentage coverage and yellow percentage coverage) alongside habitat type (terrestrial, arboreal and aquatic) and daily activity (diurnal, cathemeral and nocturnal) in the 458 snake species included in our study spanning major Colubroidae families (Colubridae, Cylindriphiidae, Lamprophiidae, Sibynophiidae, Elapidae, Viperidae).
**Table S1.** Mode of Posterior Estimates and 95% credible intervals (CI) for the posterior distributions for the red percentage coverage model which looks at the relationship between red percentage coverage and the presence of venomous traits (present, absent), along with banded patterning, daily activity, habitat type and prey visual system. The explanatory variables include the presence of venomous traits; banded patterning; daily activity; habitat type and prey visual system. The random terms associated with phylogenetic relatedness (Phylogeny (*h* ^2^)) and residual variation (Residual) are also presented. Significant values are deemed to be those with 95% of the posterior estimate above or below zero. Overall, there are 458 species in this model (including mimicking species) run across 100 randomly selected phylogenies.
**Table S2.** Mode of Posterior Estimates and 95% credible intervals (CI) for the posterior distributions for the yellow percentage coverage model which looks at the relationship between yellow percentage coverage and the presence of venomous traits (presence, absence), along with banded patterning, daily activity, habitat type and prey visual system. The explanatory variables include the presence of venomous traits; banded patterning; daily activity; habitat type and prey visual system. The random terms associated with phylogenetic relatedness (Phylogeny (*h* ^2^)) and residual variation (Residual) are also presented. Significant values are deemed to be those with 95% of the posterior estimate above or below zero. Overall, there are 458 species in this model (including mimicking species) run across 100 randomly selected phylogenies.
**Table S3.** Mode of Posterior Estimates and 95% credible intervals (CI) for the posterior distributions for the conspicuousness model which looks at the relationship between conspicuousness (measured with CDM values) and venom potency (measured in LD50 mg), along with banded patterning, daily activity, habitat type and prey visual system. The explanatory variables include venom potency; banded patterning; daily activity; habitat type and prey visual system. The random terms associated with phylogenetic relatedness (Phylogeny (*h* ^2^)) and residual variation (Residual) are also presented. Significant values are deemed to be those with 95% of the posterior estimate above or below zero. Overall, there are 88 species in this model run across 100 randomly selected phylogenies.
**Table S4.** Mode of Posterior Estimates and 95% credible intervals (CI) for the posterior distributions for the red percentage coverage model which looks at the relationship between red percentage coverage and venom potency (measured in LD50 mg), along with banded patterning, daily activity, habitat type and prey visual system. The explanatory variables include venom potency; banded patterning; daily activity; habitat type and prey visual system. The random terms associated with phylogenetic relatedness (Phylogeny (*h* ^2^)) and residual variation (Residual) are also presented. Significant values are deemed to be those with 95% of the posterior estimate above or below zero. Overall, there are 88 species in this model run across 100 randomly selected phylogenies.
**Table S5.** Mode of Posterior Estimates and 95% Credible Intervals (CI) for the posterior distributions for the yellow percentage coverage model which looks at the relationship between yellow percentage coverage and venom potency (measured in LD50 mg), along with banded patterning, daily activity, habitat type and prey visual system. The explanatory variables include venom potency; banded patterning; daily activity; habitat type and prey visual system. The random terms associated with phylogenetic relatedness (Phylogeny (*h* ^2^)) and residual variation (Residual) are also presented. Significant values are deemed to be those with 95% of the posterior estimate above or below zero. Overall, there are 88 species in this model run across 100 randomly selected phylogenies.
**Table S6.** Mode of Posterior Estimates and 95% credible intervals (CI) for the posterior distributions for the conspicuousness model which looks at the relationship between conspicuousness (measured with CDM values) and the defensive property of venom (early pain inducement), along 0.5 with banded patterning, daily activity, habitat type and prey visual system. The explanatory variables include the defensive property of venom; banded patterning; daily activity; habitat type and prey visual system. The random terms associated with phylogenetic relatedness (Phylogeny (*h* ^2^)) and residual variation (Residual) are also presented. Significant values are deemed to be those with 95% of the posterior estimate above or below zero. Overall, there are 458 species in this model run across 100 randomly selected phylogenies.
**Table S7.** Mode of Posterior Estimates and 95% credible intervals (CI) for the posterior distributions for the red percentage coverage model which looks at the relationship between red percentage coverage and the defensive property of venom (early pain inducement), along with banded patterning, daily activity, habitat type and prey visual system. The explanatory variables include the defensive property of venom; banded patterning; daily activity; habitat type and prey visual system. The random terms associated with phylogenetic relatedness (Phylogeny (*h* ^2^)) and residual variation (Residual) are also presented. Significant values are deemed to be those with 95% of the posterior estimate above or below zero. Overall, there are 458 species in this model run across 100 randomly selected phylogenies.
**Table S8.** Mode of Posterior Estimates and 95% Credible Intervals (CI) for the posterior distributions for the yellow percentage coverage model which looks at the relationship between yellow percentage coverage and the defensive property of venom (early pain inducement), along with banded patterning, daily activity, habitat type and prey visual system. The explanatory variables include the defensive property of venom; banded patterning; daily activity; habitat type and prey visual system. The random terms associated with phylogenetic relatedness (Phylogeny (*h* ^2^)), and residual variation (Residual) are also presented. Significant values are deemed to be those with 95% of the posterior estimate above or below zero. Overall, there are 458 species in this model run across 100 randomly selected phylogenies.
**Table S9.** Mode of Posterior Estimates and 95% credible intervals (CI) for the posterior distributions for the conspicuousness model which looks at the relationship between conspicuousness (measured with CDM values) and the presence of venomous traits (present, absent), along with banded patterning, daily activity, habitat type and prey visual system. The explanatory variables include the presence of venomous traits; banded patterning; daily activity; habitat type and prey visual system. The random terms associated with phylogenetic relatedness (Phylogeny (*h* ^2^)), and residual variation (Residual) are also presented. Significant values are deemed to be those with 95% of the posterior estimate above or below zero. Overall, there are 443 species (excluding mimicking species) in this model run across 100 randomly selected phylogenies.
**Table S10.** Mode of Posterior Estimates and 95% credible intervals (CI) for the posterior distributions for the conspicuousness model which looks at the relationship between red percentage coverage and the presence of venomous traits (present, absent), along with banded patterning, daily activity, habitat type and prey visual system. The explanatory variables include the presence of venomous traits; banded patterning; daily activity; habitat type and prey visual system. The random terms associated with phylogenetic relatedness (Phylogeny (*h* ^2^)), and residual variation (Residual) are also presented. Significant values are deemed to be those with 95% of the posterior estimate above or below zero. Overall, there are 443 species (excluding mimicking species) in this model run across 100 randomly selected phylogenies.
**Table S11.** Mode of Posterior Estimates and 95% credible intervals (CI) for the posterior distributions for the conspicuousness model which looks at the relationship between yellow percentage coverage and the presence of venomous traits (present, absent), along with banded patterning, daily activity, habitat type and prey visual system. The explanatory variables include the presence of venomous traits; banded patterning; daily activity; habitat type and prey visual system. The random terms associated with phylogenetic relatedness (Phylogeny (*h* ^2^)), and residual variation (Residual) are also presented. Significant values are deemed to be those with 95% of the posterior estimate above or below zero. Overall, there are 443 species (excluding mimicking species) in this model run across 100 randomly selected phylogenies.
**Figure S2.** Posterior distributions for the conspicuousness models which tests the relationship between conspicuousness (measured with CDM values) and the presence of venomous traits (present, absent), along with banded patterning, daily activity, habitat type and prey visual system.
**Figure S3.** Posterior distributions for the conspicuousness models which tests the relationship between red percentage coverage and the presence of venomous traits (present, absent), along with banded patterning, daily activity, habitat type and prey visual system.
**Figure S4.** Posterior distributions for the conspicuousness models which tests the relationship between yellow percentage coverage and the presence of venomous traits (present, absent), along with banded patterning, daily activity, habitat type and prey visual system.
**Figure S5.** Posterior distributions for the conspicuousness models which tests the relationship between conspicuousness (measured with CDM values) and venom potency (measured in LD50 mg), along with banded patterning, daily activity, habitat type and prey visual system.
**Figure S6.** Posterior distributions for the conspicuousness models which tests the relationship between red percentage coverage and venom potency (measured in LD50 mg), along with banded patterning, daily activity, habitat type and prey visual system.
**Figure S7.** Posterior distributions for the conspicuousness models which tests the relationship between yellow percentage coverage and venom potency (measured in LD50 mg), along with banded patterning, daily activity, habitat type and prey visual system.
**Figure S8.** Posterior distributions for the conspicuousness models which tests the relationship between conspicuousness (measured with CDM values) and the defensive property of venom (early pain inducement), along with banded patterning, daily activity, habitat type and prey visual system.
**Figure S9.** Posterior distributions for the conspicuousness models which tests the relationship between red percentage coverage and the defensive property of venom (early pain inducement), along with banded patterning, daily activity, habitat type and prey visual system.
**Figure S10.** Posterior distributions for the conspicuousness models which tests the relationship between yellow percentage coverage and the defensive property of venom (early pain inducement), along with banded patterning, daily activity, habitat type and prey visual system.
**Figure S11.** Posterior distributions for the conspicuousness models excluding known mimic species which tests the relationship between conspicuousness (measured with CDM values) and the presence of venomous traits (present, absent), along with banded patterning, daily activity, habitat type and prey visual system.
**Figure S12.** Posterior distributions for the conspicuousness models excluding known mimic species which tests the relationship between red percentage coverage and the presence of venomous traits (present, absent), along with banded patterning, daily activity, habitat type and prey visual system.
**Figure S13.** Posterior distributions for the conspicuousness models excluding known mimic species which tests the relationship between yellow percentage coverage and the presence of venomous traits (present, absent), along with banded patterning, daily activity, habitat type and prey visual system.
**Figure S14.** Posterior distributions for the conspicuousness model which tests the relationship conspicuousness (measured with CDM values), red percentage coverage and yellow percentage coverage.
**Figure S15.** Confidence intervals plots for the models which (a) test the relationship between conspicuousness (measured with CDM values) and the presence of venomous traits (present, absent), (b) test the relationship between red percentage coverage and the presence of venomous traits (present, absent) and (c) test the relationship between yellow percentage coverage and the presence of venomous traits (present, absent).
**Figure S16.** Confidence intervals plots for the models which (a) test the relationship between conspicuousness (measured with CDM values) and venom potency (measured in LD50 mg), (b) test the relationship between red percentage coverage and venom potency (measured in LD50 mg), and (c) test the relationship between yellow percentage coverage and venom potency (measured in LD50 mg).
**Figure S17.** Confidence intervals plots for the models which (a) test the relationship between conspicuousness (measured with CDM values) and the defensive property of venom (early pain inducement), (b) test the relationship between red percentage coverage and the defensive property of venom (early pain inducement) and (c) test the relationship between yellow percentage coverage and the defensive property of venom (early pain inducement).
**Figure S18.** Confidence intervals plots for the models which (a) test the relationship between conspicuousness (measured with CDM values) and the presence of venomous traits (present, absent) excluding known mimic species, (b) test the relationship between red percentage coverage and the presence of venomous traits (present, absent) excluding known mimic species and (c) test the relationship between yellow percentage coverage and the presence of venomous traits (present, absent) excluding known mimic species.
**Figure S19.** Confidence intervals plots for the model which tests the relationship conspicuousness (measured with CDM values), red percentage coverage and yellow percentage coverage.
**Table S12.** Mode of Prediction and 95% confidence intervals (CI) for the conspicuousness model which tests the relationship between conspicuousness (measured with CDM values) and the presence of venomous traits (present, absent), along with banded patterning, daily activity, habitat type and prey visual system.
**Table S13.** Mode of Prediction and 95% confidence intervals (CI) for the conspicuousness model which tests the relationship between red percentage coverage and the presence of venomous traits (present, absent), along with banded patterning, daily activity, habitat type and prey visual system.
**Table S14.** Mode of Prediction and 95% confidence intervals (CI) for the conspicuousness model which tests the relationship between yellow percentage coverage and the presence of venomous traits (present, absent), along with banded patterning, daily activity, habitat type and prey visual system.
**Table S15.** Mode of Prediction and 95% confidence intervals (CI) for the conspicuousness model which tests the relationship between conspicuousness (measured with CDM values) and venom potency (measured in LD50 mg), along with banded patterning, daily activity, habitat type and prey visual system.
**Table S16.** Mode of Prediction and 95% confidence intervals (CI) for the conspicuousness model which tests the relationship between red percentage coverage and venom potency (measured in LD50 mg), along with banded patterning, daily activity, habitat type and prey visual system.
**Table S17.** Mode of Prediction and 95% confidence intervals (CI) for the conspicuousness model which tests the relationship between yellow percentage coverage and venom potency (measured in LD50 mg), along with banded patterning, daily activity, habitat type and prey visual system.
**Table S18.** Mode of Prediction and 95% confidence intervals (CI) for the conspicuousness model which tests the relationship between conspicuousness (measured with CDM values) and the defensive property of venom (early pain inducement), along with banded patterning, daily activity, habitat type and prey visual system.
**Table S19.** Mode of Prediction and 95% confidence intervals (CI) for the conspicuousness model which tests the relationship between red percentage coverage and the defensive property of venom (early pain inducement), along with banded patterning, daily activity, habitat type and prey visual system.
**Table S20.** Mode of Prediction and 95% confidence intervals (CI) for the conspicuousness model which tests the relationship between yellow percentage coverage and the defensive property of venom (early pain inducement), along with banded patterning, daily activity, habitat type and prey visual system.
**Table S21.** Mode of Prediction and 95% confidence intervals (CI) for the conspicuousness model excluding known mimic species which tests the relationship between conspicuousness (measured with CDM values) and the presence of venomous traits (present, absent), along with banded patterning, daily activity, habitat type and prey visual system.
**Table S22.** Mode of Prediction and 95% confidence intervals (CI) for the conspicuousness model excluding known mimic species which tests the relationship between red percentage coverage and the presence of venomous traits (present, absent), along with banded patterning, daily activity, habitat type and prey visual system.
**Table S23.** Mode of Prediction and 95% confidence intervals (CI) for the conspicuousness model excluding known mimic species which tests the relationship between yellow percentage coverage and the presence of venomous traits (present, absent), along with banded patterning, daily activity, habitat type and prey visual system.
**Table S24.** Mode of Prediction and 95% confidence intervals (CI) for the conspicuousness model which tests the relationship conspicuousness (measured with CDM values), red percentage coverage and yellow percentage coverage.

## Data Availability

All data and code are available on Figshare (https://figshare.com/s/1b0175864ff0b54ad304).
