# Supplementary material for: Contextually conspicuous species are more likely to be venomous: Habitat‐dependent aposematic colouration found throughout snakes
Source: J Anim Ecol. 2026 Oct 4;95(11):2322–34. doi: 10.1111/1365-2656.70351 (PMC13731835; doi:10.1111/1365-2656.70351)
Supplement: Supplementary file 1 [file JANE-95-2322-s001.docx]

**Supplementary**

**Figure S1.** Phylogeny representing the diversity of conspicuousness (measured with CDM values, red percentage coverage and yellow percentage coverage) alongside habitat type (terrestrial, arboreal and aquatic) and daily activity (diurnal, cathemeral and nocturnal) in the 458 snake species included in our study spanning major Colubroidae families (Colubridae, Cylindriphiidae, Lamprophiidae, Sibynophiidae, Elapidae, Viperidae).

Table S1: Mode of Posterior Estimates and 95% Credible Intervals (CI) for the posterior distributions for the red percentage coverage model which looks at the relationship between red percentage coverage and the presence of venomous traits (present, absent), along with banded patterning, daily activity, habitat type and prey visual system. The explanatory variables include the presence of venomous traits; banded patterning; daily activity; habitat type and prey visual system. The random terms associated with phylogenetic relatedness (Phylogeny (h^2^)), and residual variation (Residual) are also presented. Significant values are deemed to be those with 95% of the posterior estimate above or below zero. Overall there are 458 species in this model (including mimicking species) run across 100 randomly selected phylogenies. Model estimates were considered significant when the 95% credible intervals (CI) did not cross zero.

|  | **Mode** | | **Lower 95% C.I.** | **Upper 95% C.I.** |
| --- | --- | --- | --- | --- |
| **Fixed terms** |  | |  |  |
| Intercept | | 0.563 | -8.437 | 9.715 |
| Venomous (Present) | | 1.671 | 0.269 | 3.051 |
| **Banded patterning (Present)** | | **2.416** | **0.677** | **4.092** |
| Daily activity (Cathemeral) | | 0.009 | -1.291 | 1.306 |
| Daily activity (Nocturnal) | | -0.911 | -2.186 | 0.333 |
| Habitat Type(Arboreal) | | 0.078 | -1.312 | 1.472 |
| Habitat Type(Aquatic) | | -1.289 | -3.527 | 1.105 |
| Prey visual system (Complex) | | 0.087 | -1.284 | 1.469 |
| **Random terms** |  | |  |  |
| Residual Variance | 19.121 | | 17.845 | 20.600 |
| Phylogenetic Variance | 78.970 | | 59.888 | 101.092 |
| h^2^ | 0.822 | |  |  |

Table S2: Mode of Posterior Estimates and 95% Credible Intervals (CI) for the posterior distributions for the yellow percentage coverage model which looks at the relationship between yellow percentage coverage and the presence of venomous traits (presence, absence), along with banded patterning, daily activity, habitat type and prey visual system. The explanatory variables include the presence of venomous traits; banded patterning; daily activity; habitat type and prey visual system. The random terms associated with phylogenetic relatedness (Phylogeny (h^2^)), and residual variation (Residual) are also presented. Significant values are deemed to be those with 95% of the posterior estimate above or below zero. Overall there are 458 species in this model (including mimicking species) run across 100 randomly selected phylogenies. Model estimates were considered significant when the 95% credible intervals (CI) did not cross zero.

|  | **Mode** | **Lower 95% C.I.** | **Upper 95% C.I.** |
| --- | --- | --- | --- |
| **Fixed terms** |  |  |  |
| Intercept | -0.096 | -0.922 | 0.634 |
| Venomous (Present) | 0.096 | -0.192 | 0.406 |
| Banded patterning (Present) | 0.004 | -0.398 | 0.369 |
| **Daily activity (Cathemeral)** | **0.384** | **0.051** | **0.715** |
| Daily activity (Nocturnal) | 0.111 | -0.143 | 0.379 |
| **Habitat Type(Arboreal)** | **0.738** | **0.452** | **1.007** |
| **Habitat Type(Aquatic)** | **0.423** | **0.014** | **0.820** |
| Prey visual system (Complex) | 0.126 | -0.181 | 0.436 |
| **Random terms** |  |  |  |
| Residual Variance | 6.096 | 5.694 | 6.495 |
| Phylogenetic Variance | 0.002 | -0.001 | 0.972 |
| h^2^ | 0.0004 |  |  |

Table S3: Mode of Posterior Estimates and 95% Credible Intervals (CI) for the posterior distributions for the conspicuousness model which looks at the relationship between conspicuousness (measured with CDM values) and venom potency (measured in LD50 mg), along with banded patterning, daily activity, habitat type and prey visual system. The explanatory variables include venom potency; banded patterning; daily activity; habitat type and prey visual system. The random terms associated with phylogenetic relatedness (Phylogeny (h^2^)), and residual variation (Residual) are also presented. Significant values are deemed to be those with 95% of the posterior estimate above or below zero. Overall there are 88 species in this model run across 100 randomly selected phylogenies.

|  | **Mode** | | **Lower 95% C.I.** | **Upper 95% C.I.** |
| --- | --- | --- | --- | --- |
| **Fixed terms** |  | |  |  |
| Intercept | | 0.262 | 0.147 | 3.732 |
| Venom potency (LD_50_) | | -0.0000007 | -0.00007 | 6.132 |
| Banded patterning (Present) | | 3.378 | -0.039 | 1.055 |
| Daily activity (Cathemeral) | | -0.016 | -0.051 | 2.119 |
| Daily activity (Nocturnal) | | -0.015 | -0.058 | 2.761 |
| Habitat Type(Arboreal) | | -0.025 | -0.079 | 2.895 |
| Habitat Type(Aquatic) | | 1.944 | -0.037 | 7.553 |
| Prey visual system (Complex) | | -0.006 | -0.051 | 7.553 |
| **Random terms** |  | |  |  |
| Residual Variance | 0.014 | | 0.013 | 1.446 |
| Phylogenetic Variance | 0.013 | | 0.008 | 2.166 |
| h^2^ | 0.545 | |  |  |

Table S4: Mode of Posterior Estimates and 95% Credible Intervals (CI) for the posterior distributions for the red percentage coverage model which looks at the relationship between red percentage coverage and venom potency (measured in LD50 mg), along with banded patterning, daily activity, habitat type and prey visual system. The explanatory variables include venom potency; banded patterning; daily activity; habitat type and prey visual system. The random terms associated with phylogenetic relatedness (Phylogeny (h^2^)), and residual variation (Residual) are also presented. Significant values are deemed to be those with 95% of the posterior estimate above or below zero. Overall there are 88 species in this model run across 100 randomly selected phylogenies.

|  | **Mode** | **Lower 95% C.I.** | **Upper 95% C.I.** |
| --- | --- | --- | --- |
| **Fixed terms** |  |  |  |
| Intercept | -0.293 | -3.602 | 2.968 |
| Venom potency (LD_50_) | -0.00002 | -0.002 | 0.001 |
| Banded patterning (Present) | 1.059 | -1.004 | 3.160 |
| Daily activity (Cathemeral) | 0.085 | -0.909 | 1.092 |
| Daily activity (Nocturnal) | 0.300 | -0.894 | 1.504 |
| Habitat Type(Arboreal) | -0.055 | -1.542 | 1.435 |
| Habitat Type(Aquatic) | 0.743 | -0.858 | 2.372 |
| Prey visual system (Complex) | 0. 169 | -1.083 | 1.442 |
| **Random terms** |  |  |  |
| Residual Variance | 7.047 | 6.660 | 7.452 |
| Phylogenetic Variance | 0.118 | 7.675 | 18.569 |
| h^2^ | 0.681 |  |  |

Table S5: Mode of Posterior Estimates and 95% Credible Intervals (CI) for the posterior distributions for the yellow percentage coverage model which looks at the relationship between yellow percentage coverage and venom potency (measured in LD50 mg), along with banded patterning, daily activity, habitat type and prey visual system. The explanatory variables include venom potency; banded patterning; daily activity; habitat type and prey visual system. The random terms associated with phylogenetic relatedness (Phylogeny (h^2^)), and residual variation (Residual) are also presented. Significant values are deemed to be those with 95% of the posterior estimate above or below zero. Overall there are 88 species in this model run across 100 randomly selected phylogenies. Model estimates were considered significant when the 95% credible intervals (CI) did not cross zero.

|  | **Mode** | **Lower 95% C.I.** | **Upper 95% C.I.** |
| --- | --- | --- | --- |
| **Fixed terms** |  |  |  |
| Intercept | -0.029 | -0.873 | 0.804 |
| Venom potency (LD_50_) | -1.887 | -0.0004 | 0.0003 |
| **Banded patterning (Present)** | **0.582** | **0.038** | **1.129** |
| Daily activity (Cathemeral) | 0.011 | -0.241 | 0.269 |
| Daily activity (Nocturnal) | 0.008 | -0.299 | -0.097 |
| Habitat Type(Arboreal) | 0.215 | -0.176 | 0.592 |
| Habitat Type(Aquatic) | 0.012 | -0.399 | 0.427 |
| Prey visual system (Complex) | 0.132 | -0.188 | 0.452 |
| **Random terms** |  |  |  |
| Residual Variance | 0.448 | 0.424 | 0.474 |
| Phylogenetic Variance | 0.759 | 0.511 | 1.207 |
| h^2^ | 0.685 |  |  |

Table S6: Mode of Posterior Estimates and 95% Credible Intervals (CI) for the posterior distributions for the conspicuousness model which looks at the relationship between conspicuousness (measured with CDM values) and the defensive property of venom (early pain inducement), along 0.5with banded patterning, daily activity, habitat type and prey visual system. The explanatory variables include the defensive property of venom; banded patterning; daily activity; habitat type and prey visual system. The random terms associated with phylogenetic relatedness (Phylogeny (h^2^)), and residual variation (Residual) are also presented. Significant values are deemed to be those with 95% of the posterior estimate above or below zero. Overall there are 458 species in this model run across 100 randomly selected phylogenies.

|  | **Mode** | **Lower 95% C.I.** | **Upper 95% C.I.** |
| --- | --- | --- | --- |
| **Fixed terms** |  |  |  |
| Intercept | 0.259 | 0.192 | 0.322 |
| Defensive venom (Present) | 0.010 | -0.011 | 0.029 |
| Banded patterning (Present) | 0.021 | -0.026 | 0.069 |
| Daily activity (Cathemeral) | -0.012 | -0.040 | 0.015 |
| Daily activity (Nocturnal) | -0.015 | -0.039 | 0.008 |
| Habitat Type(Arboreal) | 0.003 | -0.025 | 0.028 |
| Habitat Type(Aquatic) | -0.0005 | -0.049 | 0.048 |
| Prey visual system (Complex) | -0.007 | -0.047 | 0.033 |
| **Random terms** |  |  |  |
| Residual Variance | 0.014 | 0.013 | 0.016 |
| Phylogenetic Variance | 0.002 | 0.0005 | 0.004 |
| h^2^ | 0.105 |  |  |

Table S7: Mode of Posterior Estimates and 95% Credible Intervals (CI) for the posterior distributions for the red percentage coverage model which looks at the relationship between red percentage coverage and the defensive property of venom (early pain inducement), along with banded patterning, daily activity, habitat type and prey visual system. The explanatory variables include the defensive property of venom; banded patterning; daily activity; habitat type and prey visual system. The random terms associated with phylogenetic relatedness (Phylogeny (h^2^)), and residual variation (Residual) are also presented. Significant values are deemed to be those with 95% of the posterior estimate above or below zero. Overall there are 458 species in this model run across 100 randomly selected phylogenies. Model estimates were considered significant when the 95% credible intervals (CI) did not cross zero.

|  | **Mode** | **Lower 95% C.I.** | **Upper 95% C.I.** |
| --- | --- | --- | --- |
| **Fixed terms** |  |  |  |
| Intercept | -0.126 | -1.842 | 1.512 |
| Defensive venom (Present) | -0.604 | -1.230 | 0.016 |
| **Banded patterning (Present)** | **8.646** | **7.025** | **10.055** |
| Daily activity (Cathemeral) | 0.524 | -0.328 | 1.346 |
| Daily activity (Nocturnal) | -0.063 | -0.752 | 0.664 |
| Habitat Type(Arboreal) | -0.442 | -1.235 | 0.389 |
| Habitat Type(Aquatic) | -0.260 | -1.729 | 1.167 |
| Prey visual system (Complex) | 0.740 | -0.489 | 2.044 |
| **Random terms** |  |  |  |
| Residual Variance | 16.347 | 14.401 | 18.176 |
| Phylogenetic Variance | 0.026 | -0.004 | 5.308 |
| h^2^ | 0.0004 |  |  |

Table S8: Mode of Posterior Estimates and 95% Credible Intervals (CI) for the posterior distributions for the yellow percentage coverage model which looks at the relationship between yellow percentage coverage and the defensive property of venom (early pain inducement), along with banded patterning, daily activity, habitat type and prey visual system. The explanatory variables include the defensive property of venom; banded patterning; daily activity; habitat type and prey visual system. The random terms associated with phylogenetic relatedness (Phylogeny (h^2^)), and residual variation (Residual) are also presented. Significant values are deemed to be those with 95% of the posterior estimate above or below zero. Overall there are 458 species in this model run across 100 randomly selected phylogenies. Model estimates were considered significant when the 95% credible intervals (CI) did not cross zero.

|  | **Mode** | **Lower 95% C.I.** | **Upper 95% C.I.** |
| --- | --- | --- | --- |
| **Fixed terms** |  |  |  |
| Intercept | -0.252 | -0.832 | 0.330 |
| Defensive venom (Present) | -01.09 | -0.355 | 0.141 |
| **Banded patterning (Present)** | **1.131** | **0.598** | **1.670** |
| Daily activity (Cathemeral) | 0.272 | -0.061 | 0.601 |
| Daily activity (Nocturnal) | 0.185 | -0.085 | 0.459 |
| **Habitat Type(Arboreal)** | **0.729** | **0.407** | **1.040** |
| Habitat Type(Aquatic) | 0.025 | -0.531 | 0.584 |
| Prey visual system (Complex) | 0.280 | -0.212 | 0.766 |
| **Random terms** |  |  |  |
| Residual Variance | 2.705 | 2.448 | 2.988 |
| Phylogenetic Variance | 0.002 | -0.0003 | 0.223 |
| h^2^ | 0.0004 |  |  |

Table S9: Mode of Posterior Estimates and 95% Credible Intervals (CI) for the posterior distributions for the conspicuousness model which looks at the relationship between conspicuousness (measured with CDM values) and the presence of venomous traits (present, absent), along with banded patterning, daily activity, habitat type and prey visual system. The explanatory variables include the presence of venomous traits; banded patterning; daily activity; habitat type and prey visual system. The random terms associated with phylogenetic relatedness (Phylogeny (h^2^)), and residual variation (Residual) are also presented. Significant values are deemed to be those with 95% of the posterior estimate above or below zero. Overall there are 443 species (excluding mimicking species) in this model run across 100 randomly selected phylogenies. Model estimates were considered significant when the 95% credible intervals (CI) did not cross zero.

|  | **Mode** | | **Lower 95% C.I.** | **Upper 95% C.I.** |
| --- | --- | --- | --- | --- |
| **Fixed terms** |  | |  |  |
| Intercept | | 0.219 | 0.173 | 0.264 |
| **Venomous (Present)** | | **0.025** | **0.007** | **0.043** |
| Banded patterning (Present) | | 0.020 | -0.005 | 0.044 |
| Daily activity (Cathemeral) | | -0.012 | -0.030 | 0.007 |
| Daily activity (Nocturnal) | | -0.012 | -0.029 | 0.003 |
| Habitat Type(Arboreal) | | 0.005 | -0.013 | 0.022 |
| Habitat Type(Aquatic) | | 0.009 | -0.016 | 0.034 |
| Prey visual system (Complex) | | 0.015 | -0.004 | 0.032 |
| **Random terms** |  | |  |  |
| Residual Variance | 0.015 | | 0.014 | 0.016 |
| Phylogenetic Variance | 0.002 | | 0.001 | 0.003 |
| h^2^ | 0.121 | |  |  |

Table S10: Mode of Posterior Estimates and 95% Credible Intervals (CI) for the posterior distributions for the conspicuousness model which looks at the relationship between red percentage coverage and the presence of venomous traits (present, absent), along with banded patterning, daily activity, habitat type and prey visual system. The explanatory variables include the presence of venomous traits; banded patterning; daily activity; habitat type and prey visual system. The random terms associated with phylogenetic relatedness (Phylogeny (h^2^)), and residual variation (Residual) are also presented. Significant values are deemed to be those with 95% of the posterior estimate above or below zero. Overall there are 443 species (excluding mimicking species) in this model run across 100 randomly selected phylogenies. Model estimates were considered significant when the 95% credible intervals (CI) did not cross zero.

|  | **Mode** | **Lower 95% C.I.** | **Upper 95% C.I.** |
| --- | --- | --- | --- |
| **Fixed terms** |  |  |  |
| Intercept | 0.279 | -5.773 | 6.340 |
| **Venomous (Present)** | **1.745** | **0.286** | **3.207** |
| **Banded patterning (Present)** | **2.299** | **0.274** | **4.099** |
| Daily activity (Cathemeral) | 0.001 | -1.320 | 1.303 |
| Daily activity (Nocturnal) | -0.748 | -2.065 | 0.475 |
| Habitat Type(Arboreal) | 0.079 | -1.354 | 1.603 |
| Habitat Type(Aquatic) | -1.313 | -3.586 | 1.147 |
| Prey visual system (Complex) | -0.004 | -1.380 | 1.406 |
| **Random terms** |  |  |  |
| Residual Variance | 18.341 | 17.077 | 19.766 |
| Phylogenetic Variance | 58.946 | 46.209 | 82.299 |
| h^2^ | 0.801 |  |  |

Table S11: Mode of Posterior Estimates and 95% Credible Intervals (CI) for the posterior distributions for the conspicuousness model which looks at the relationship between yellow percentage coverage and the presence of venomous traits (present, absent), along with banded patterning, daily activity, habitat type and prey visual system. The explanatory variables include the presence of venomous traits; banded patterning; daily activity; habitat type and prey visual system. The random terms associated with phylogenetic relatedness (Phylogeny (h^2^)), and residual variation (Residual) are also presented. Significant values are deemed to be those with 95% of the posterior estimate above or below zero. Overall there are 443 species (excluding mimicking species) in this model run across 100 randomly selected phylogenies. Model estimates were considered significant when the 95% credible intervals (CI) did not cross zero.

|  | **Mode** | **Lower 95% C.I.** | **Upper 95% C.I.** |
| --- | --- | --- | --- |
| **Fixed terms** |  |  |  |
| Intercept | -0.120 | -0.760 | 0.463 |
| Venomous (Present) | 0.125 | -0.187 | 0.454 |
| Banded patterning (Present) | -0.048 | -0.511 | 0.364 |
| **Daily activity (Cathemeral)** | **0.388** | **0.046** | **0.730** |
| Daily activity (Nocturnal) | 0.121 | -0.146 | 0.402 |
| **Habitat Type(Arboreal)** | **0.727** | **0.427** | **1.011** |
| Habitat Type(Aquatic) | 0.427 | -0.005 | 0.829 |
| Prey visual system (Complex) | 0.119 | -0.203 | 0.438 |
| **Random terms** |  |  |  |
| Residual Variance | 6.229 | 5.814 | 6.654 |
| Phylogenetic Variance | 0.001 | -0.001 | 0.821 |
| h^2^ | 0.0004 |  |  |

**Figure S2.** Posterior distributions for the conspicuousness models which tests the relationship between conspicuousness (measured with CDM values) and the presence of venomous traits (present, absent), along with banded patterning, daily activity, habitat type and prey visual system.

**Figure S3.** Posterior distributions for the conspicuousness models which tests the relationship between red percentage coverage and the presence of venomous traits (present, absent), along with banded patterning, daily activity, habitat type and prey visual system.

**Figure S4.** Posterior distributions for the conspicuousness models which tests the relationship between yellow percentage coverage and the presence of venomous traits (present, absent), along with banded patterning, daily activity, habitat type and prey visual system.

**Figure S5.** Posterior distributions for the conspicuousness models which tests the relationship between conspicuousness (measured with CDM values) and venom potency (measured in LD50 mg), along with banded patterning, daily activity, habitat type and prey visual system.

**Figure S6.** Posterior distributions for the conspicuousness models which tests the relationship between red percentage coverage and venom potency (measured in LD50 mg), along with banded patterning, daily activity, habitat type and prey visual system.

**Figure S7.** Posterior distributions for the conspicuousness models which tests the relationship between yellow percentage coverage and venom potency (measured in LD50 mg), along with banded patterning, daily activity, habitat type and prey visual system.

**Figure S8.** Posterior distributions for the conspicuousness models which tests the relationship between conspicuousness (measured with CDM values) and the defensive property of venom (early pain inducement), along with banded patterning, daily activity, habitat type and prey visual system.

**Figure S9.** Posterior distributions for the conspicuousness models which tests the relationship between red percentage coverage and the defensive property of venom (early pain inducement), along with banded patterning, daily activity, habitat type and prey visual system.

**Figure S10.** Posterior distributions for the conspicuousness models which tests the relationship between yellow percentage coverage and the defensive property of venom (early pain inducement), along with banded patterning, daily activity, habitat type and prey visual system.

**Figure S11.** Posterior distributions for the conspicuousness models excluding known mimic species which tests the relationship between conspicuousness (measured with CDM values) and the presence of venomous traits (present, absent), along with banded patterning, daily activity, habitat type and prey visual system.

**Figure S12.** Posterior distributions for the conspicuousness models excluding known mimic species which tests the relationship between red percentage coverage and the presence of venomous traits (present, absent), along with banded patterning, daily activity, habitat type and prey visual system.

**Figure S13.** Posterior distributions for the conspicuousness models excluding known mimic species which tests the relationship between yellow percentage coverage and the presence of venomous traits (present, absent), along with banded patterning, daily activity, habitat type and prey visual system.

**Figure S14.** Posterior distributions for the conspicuousness model which tests the relationship conspicuousness (measured with CDM values), red percentage coverage and yellow percentage coverage.

**Figure S15.** Confidence Intervals plots for the models which a) test the relationship between conspicuousness (measured with CDM values) and the presence of venomous traits (present, absent), b) test the relationship between red percentage coverage and the presence of venomous traits (present, absent), and c) test the relationship between yellow percentage coverage and the presence of venomous traits (present, absent).

**Figure S16.** Confidence Intervals plots for the models which a) test the relationship between conspicuousness (measured with CDM values) and venom potency (measured in LD50 mg), b) test the relationship between red percentage coverage and venom potency (measured in LD50 mg), and c) test the relationship between yellow percentage coverage and venom potency (measured in LD50 mg).

**Figure S17.** Confidence Intervals plots for the models which a) test the relationship between conspicuousness (measured with CDM values) and the defensive property of venom (early pain inducement), b) test the relationship between red percentage coverage and the defensive property of venom (early pain inducement), and c) test the relationship between yellow percentage coverage and the defensive property of venom (early pain inducement).

**Figure S18.** Confidence Intervals plots for the models which a) test the relationship between conspicuousness (measured with CDM values) and the presence of venomous traits (present, absent) excluding known mimic species, b) test the relationship between red percentage coverage and the presence of venomous traits (present, absent) excluding known mimic species, and c) test the relationship between yellow percentage coverage and the presence of venomous traits (present, absent) excluding known mimic species.

**Figure S19.** Confidence Intervals plots for the model which tests the relationship conspicuousness (measured with CDM values), red percentage coverage and yellow percentage coverage.

Table S12: Mode of Prediction and 95% Confidence Intervals (CI) for the conspicuousness model which tests the relationship between conspicuousness (measured with CDM values) and the presence of venomous traits (present, absent), along with banded patterning, daily activity, habitat type and prey visual system.

|  | **Mode** | **Lower 95% C.I.** | **Upper 95% C.I.** |
| --- | --- | --- | --- |
| Venomous | 0.249 | 0. 191 | 0.311 |
| Non-Venomous | 0.230 | 0.174 | 0.295 |
| Banded Patterning (Present) | 0.268 | 0.205 | 0.330 |
| Banded patterning (Absent) | 0.248 | 0.189 | 0.309 |
| Daily Activity (Diurnal) | 0.258 | 0.197 | 0.318 |
| Daily activity (Cathemeral) | 0.244 | 0.184 | 0.306 |
| Daily activity (Nocturnal) | 0.243 | 0.187 | 0.306 |
| Habitat Type (Terrestrial) | 0.249 | 0.190 | 0.310 |
| Habitat Type(Arboreal) | 0.249 | 0.191 | 0.313 |
| Habitat Type(Aquatic) | 0.259 | 0.201 | 0.325 |
| Prey visual system (Simple) | 0.234 | 0.175 | 0.297 |
| Prey visual system (Complex) | 0.250 | 0.191 | 0.311 |

Table S13: Mode of Prediction and 95% Confidence Intervals (CI) for the conspicuousness model which tests the relationship between red percentage coverage and the presence of venomous traits (present, absent), along with banded patterning, daily activity, habitat type and prey visual system.

|  | **Mode** | **Lower 95% C.I.** | **Upper 95% C.I.** |
| --- | --- | --- | --- |
| Venomous | 2.349 | -6.414 | 11.326 |
| Non-Venomous | 0.304 | -8.992 | 8.817 |
| Banded Patterning (Present) | 4.157 | -4.672 | 13.474 |
| Banded patterning (Absent) | 2.184 | -6.586 | 11.129 |
| Daily Activity (Diurnal) | 2.693 | -6.069 | 11.549 |
| Daily activity (Cathemeral) | 2.623 | -6.489 | 11.238 |
| Daily activity (Nocturnal) | 1.751 | -6.838 | 11.031 |
| Habitat Type (Terrestrial) | 2.298 | -6.386 | 11.290 |
| Habitat Type(Arboreal) | 2.563 | -6.796 | 11.345 |
| Habitat Type(Aquatic) | 0.551 | -8.584 | 9.649 |
| Prey visual system (Simple) | 2.627 | -6.509 | 11.557 |
| Prey visual system (Complex) | 2.293 | -6.465 | 11.262 |

Table S14: Mode of Prediction and 95% Confidence Intervals (CI) for the conspicuousness model which tests the relationship between yellow percentage coverage and the presence of venomous traits (present, absent), along with banded patterning, daily activity, habitat type and prey visual system.

|  | **Mode** | **Lower 95% C.I.** | **Upper 95% C.I.** |
| --- | --- | --- | --- |
| Venomous | 0.334 | -0.274 | 0.954 |
| Non-Venomous | 0.300 | -0.369 | 0.857 |
| Banded Patterning (Present) | 0.207 | -0.513 | 0.814 |
| Banded patterning (Absent) | 0.342 | -0.260 | 0.961 |
| Daily Activity (Diurnal) | 0.237 | -0.422 | 0.786 |
| Daily activity (Cathemeral) | 0.516 | -0.107 | 1.134 |
| Daily activity (Nocturnal) | 0.321 | -0.232 | 1.007 |
| Habitat Type (Terrestrial) | 0.246 | -0.355 | 0.866 |
| Habitat Type(Arboreal) | 0.910 | 0.253 | 1.519 |
| Habitat Type(Aquatic) | 0.593 | -0.082 | 1.249 |
| Prey visual system (Simple) | 0.248 | -0.431 | 0.866 |
| Prey visual system (Complex) | 0.337 | - 0.269 | 0.956 |

Table S15: Mode of Prediction and 95% Confidence Intervals (CI) for the conspicuousness model which tests the relationship between conspicuousness (measured with CDM values) and venom potency (measured in LD50 mg), along with banded patterning, daily activity, habitat type and prey visual system.

|  | **Mode** | **Lower 95% C.I.** | **Upper 95% C.I.** |
| --- | --- | --- | --- |
| Venom Potency (LD50) | 0.239 | 0.125 | 0.358 |
| Banded Patterning (Present) | 0.287 | 0.158 | 0.428 |
| Banded patterning (Absent) | 0.244 | 0.128 | 0.364 |
| Daily Activity (Diurnal) | 0.260 | 0.142 | 0.385 |
| Daily activity (Cathemeral) | 0.238 | 0.121 | 0.355 |
| Daily activity (Nocturnal) | 0.237 | 0.122 | 0.357 |
| Habitat Type (Terrestrial) | 0.248 | 0.133 | 0.369 |
| Habitat Type(Arboreal) | 0.226 | 0.102 | 0.354 |
| Habitat Type(Aquatic) | 0.273 | 0.145 | 0.402 |
| Prey visual system (Simple) | 0.244 | 0.112 | 0.359 |
| Prey visual system (Complex) | 0.248 | 0.131 | 0.370 |

Table S16: Mode of Prediction and 95% Confidence Intervals (CI) for the conspicuousness model which tests the relationship between red percentage coverage and venom potency (measured in LD50 mg), along with banded patterning, daily activity, habitat type and prey visual system.

|  | **Mode** | **Lower 95% C.I.** | **Upper 95% C.I.** |
| --- | --- | --- | --- |
| Venom Potency (LD50) | 0.162 | -3.253 | 3.669 |
| Banded Patterning (Present) | 0.892 | -3.216 | 4.856 |
| Banded patterning (Absent) | 0.036 | -3.420 | 3.593 |
| Daily Activity (Diurnal) | 0.123 | -3.455 | 3.800 |
| Daily activity (Cathemeral) | -0.062 | -3.532 | 3.474 |
| Daily activity (Nocturnal) | 0.184 | -3.271 | 3.700 |
| Habitat Type (Terrestrial) | 0.083 | -3.411 | 3.655 |
| Habitat Type(Arboreal) | 0.154 | -3.591 | 3.837 |
| Habitat Type(Aquatic) | 0.668 | -2.908 | 4.567 |
| Prey visual system (Simple) | -0.196 | -4.166 | 3.295 |
| Prey visual system (Complex) | 0.119 | -3.387 | 3.715 |

Table S17: Mode of Prediction and 95% Confidence Intervals (CI) for the conspicuousness model which tests the relationship between yellow percentage coverage and venom potency (measured in LD50 mg), along with banded patterning, daily activity, habitat type and prey visual system.

|  | **Mode** | **Lower 95% C.I.** | **Upper 95% C.I.** |
| --- | --- | --- | --- |
| Venom Potency (LD50) | 0.107 | -0.748 | 1.002 |
| Banded Patterning (Present) | 0.729 | -0.253 | 1.742 |
| Banded patterning (Absent) | 0.107 | -0.734 | 1.024 |
| Daily Activity (Diurnal) | 0.210 | -0.637 | 1.169 |
| Daily activity (Cathemeral) | 0.088 | -0.769 | 0.976 |
| Daily activity (Nocturnal) | 0.140 | -0.722 | 1.040 |
| Habitat Type (Terrestrial) | 0.151 | -0.688 | 1.075 |
| Habitat Type(Arboreal) | 0.366 | -0.587 | 1.317 |
| Habitat Type(Aquatic) | 0.101 | -0.903 | 1.002 |
| Prey visual system (Simple) | -0.015 | -0.977 | 0.861 |
| Prey visual system (Complex) | 0.167 | -0.686 | 1.093 |

Table S18: Mode of Prediction and 95% Confidence Intervals (CI) for the conspicuousness model which tests the relationship between conspicuousness (measured with CDM values) and the defensive property of venom (early pain inducement), along with banded patterning, daily activity, habitat type and prey visual system.

|  | **Mode** | **Lower 95% C.I.** | **Upper 95% C.I.** |
| --- | --- | --- | --- |
| Defensive Venomous (Present) | 0.250 | 0.193 | 0.306 |
| Defensive Venomous (Absent) | 0.242 | 0.185 | 0.296 |
| Banded Patterning (Present) | 0.271 | 0.199 | 0.343 |
| Banded patterning (Absent) | 0.243 | 0.187 | 0.297 |
| Daily Activity (Diurnal) | 0.254 | 0.197 | 0.311 |
| Daily activity (Cathemeral) | 0.240 | 0.182 | 0.293 |
| Daily activity (Nocturnal) | 0.239 | 0.183 | 0.292 |
| Habitat Type (Terrestrial) | 0.245 | 0.188 | 0.299 |
| Habitat Type(Arboreal) | 0.247 | 0.187 | 0.302 |
| Habitat Type(Aquatic) | 0.240 | 0.169 | 0.306 |
| Prey visual system (Simple) | 0.252 | 0.184 | 0.313 |
| Prey visual system (Complex) | 0.245 | 0.188 | 0.299 |

Table S19: Mode of Prediction and 95% Confidence Intervals (CI) for the conspicuousness model which tests the relationship between red percentage coverage and the defensive property of venom (early pain inducement), along with banded patterning, daily activity, habitat type and prey visual system.

|  | **Mode** | **Lower 95% C.I.** | **Upper 95% C.I.** |
| --- | --- | --- | --- |
| Defensive Venomous (Present) | 0.562 | -0.894 | 1.959 |
| Defensive Venomous (Absent) | 1.148 | - 0.238 | 2.497 |
| Banded Patterning (Present) | 8.805 | 6.879 | 10.570 |
| Banded patterning (Absent) | 0.430 | -0.945 | 1.769 |
| Daily Activity (Diurnal) | 1.449 | 0.050 | 2.832 |
| Daily activity (Cathemeral) | 0.864 | -0.545 | 2.214 |
| Daily activity (Nocturnal) | 0.518 | -0.902 | 1.875 |
| Habitat Type (Terrestrial) | 1.031 | -0.379 | 2.368 |
| Habitat Type(Arboreal) | 0.457 | -0.920 | 1.962 |
| Habitat Type(Aquatic) | 0.313 | - 1.365 | 2.183 |
| Prey visual system (Simple) | 1.057 | -0.777 | 2.677 |
| Prey visual system (Complex) | 0.945 | -0.446 | 2.300 |

Table S20: Mode of Prediction and 95% Confidence Intervals (CI) for the conspicuousness model which tests the relationship between yellow percentage coverage and the defensive property of venom (early pain inducement), along with banded patterning, daily activity, habitat type and prey visual system.

|  | **Mode** | **Lower 95% C.I.** | **Upper 95% C.I.** |
| --- | --- | --- | --- |
| Defensive Venomous (Present) | 0.194 | -0.174 | 0.598 |
| Defensive Venomous (Absent) | 0.309 | -0.063 | 0.666 |
| Banded Patterning (Present) | 1.199 | 0.600 | 1.769 |
| Banded patterning (Absent) | 0.209 | -0.147 | 0.568 |
| Daily Activity (Diurnal) | 0.249 | -0.138 | 0.627 |
| Daily activity (Cathemeral) | 0.268 | -0.101 | 0.642 |
| Daily activity (Nocturnal) | 0.292 | -0.064 | 0.659 |
| Habitat Type (Terrestrial) | 0.187 | -0.172 | 0.548 |
| Habitat Type(Arboreal) | 0.861 | 0.432 | 1.292 |
| Habitat Type(Aquatic) | 0.153 | -0.458 | 0.772 |
| Prey visual system (Simple) | 0.186 | -0.375 | 0.780 |
| Prey visual system (Complex) | 0.274 | -0.089 | 0.637 |

Table S21: Mode of Prediction and 95% Confidence Intervals (CI) for the conspicuousness model excluding known mimic species which tests the relationship between conspicuousness (measured with CDM values) and the presence of venomous traits (present, absent), along with banded patterning, daily activity, habitat type and prey visual system.

|  | **Mode** | **Lower 95% C.I.** | **Upper 95% C.I.** |
| --- | --- | --- | --- |
| Venomous | 0.248 | 0.205 | 0.288 |
| Non-Venomous | 0.232 | 0.188 | 0.273 |
| Banded Patterning (Present) | 0.271 | 0.224 | 0.318 |
| Banded patterning (Absent) | 0.246 | 0.203 | 0.286 |
| Daily Activity (Diurnal) | 0.257 | 0.215 | 0.298 |
| Daily activity (Cathemeral) | 0.243 | 0.198 | 0.284 |
| Daily activity (Nocturnal) | 0.242 | 0.199 | 0.280 |
| Habitat Type (Terrestrial) | 0.247 | 0.204 | 0.286 |
| Habitat Type(Arboreal) | 0.252 | 0.208 | 0.294 |
| Habitat Type(Aquatic) | 0.258 | 0.212 | 0.303 |
| Prey visual system (Simple) | 0.233 | 0.188 | 0.279 |
| Prey visual system (Complex) | 0.248 | 0.205 | 0.288 |

Table S22: Mode of Prediction and 95% Confidence Intervals (CI) for the conspicuousness model excluding known mimic species which tests the relationship between red percentage coverage and the presence of venomous traits (present, absent), along with banded patterning, daily activity, habitat type and prey visual system.

|  | **Mode** | **Lower 95% C.I.** | **Upper 95% C.I.** |
| --- | --- | --- | --- |
| Venomous | 1.953 | -4.853 | 8.605 |
| Non-Venomous | 0.354 | -6.630 | 7.141 |
| Banded Patterning (Present) | 4.077 | -2.825 | 10.707 |
| Banded patterning (Absent) | 1.786 | -5.016 | 8.442 |
| Daily Activity (Diurnal) | 2.251 | -4.725 | 8.838 |
| Daily activity (Cathemeral) | 2.200 | -4.848 | 8.615 |
| Daily activity (Nocturnal) | 1.462 | -5.043 | 8.322 |
| Habitat Type (Terrestrial) | 1.966 | -4.828 | 8.628 |
| Habitat Type(Arboreal) | 1.905 | -4.978 | 8.501 |
| Habitat Type(Aquatic) | -0.007 | -7.068 | 6.694 |
| Prey visual system (Simple) | 2.445 | -4.410 | 9.175 |
| Prey visual system (Complex) | 1.908 | -4.898 | 8.559 |

Table S23: Mode of Prediction and 95% Confidence Intervals (CI) for the conspicuousness model excluding known mimic species which tests the relationship between yellow percentage coverage and the presence of venomous traits (present, absent), along with banded patterning, daily activity, habitat type and prey visual system.

|  | **Mode** | **Lower 95% C.I.** | **Upper 95% C.I.** |
| --- | --- | --- | --- |
| Venomous | 0.336 | -0.175 | 0.828 |
| Non-Venomous | 0.362 | -0.196 | 0.858 |
| Banded Patterning (Present) | 0.147 | -0.472 | 0.773 |
| Banded patterning (Absent) | 0.349 | -0.155 | 0.832 |
| Daily Activity (Diurnal) | 0.229 | -0.287 | 0.704 |
| Daily activity (Cathemeral) | 0.519 | -0.015 | 1.046 |
| Daily activity (Nocturnal) | 0.332 | -0.162 | 0.821 |
| Habitat Type (Terrestrial) | 0.250 | -0.254 | 0.741 |
| Habitat Type(Arboreal) | 0.895 | 0.342 | 1.395 |
| Habitat Type(Aquatic) | 0.602 | -0.011 | 1.135 |
| Prey visual system (Simple) | 0.244 | -0.336 | 0.815 |
| Prey visual system (Complex) | 0.340 | -0.168 | 0.829 |

Table S24: Mode of Prediction and 95% Confidence Intervals (CI) for the conspicuousness model which tests the relationship conspicuousness (measured with CDM values), red percentage coverage and yellow percentage coverage.

|  | **Mode** | **Lower 95% C.I.** | **Upper 95% C.I.** |
| --- | --- | --- | --- |
| Red Percentage coverage | 0.239 | 0.184 | 0.299 |
| Yellow Percentage coverage | 0.237 | 0.182 | 0.297 |
